# Lower pole approach in retroperitoneal laparoscopic radical nephrectomy: a new approach for the management of renal vascular pedicle

**DOI:** 10.1186/s12957-018-1324-7

**Published:** 2018-02-17

**Authors:** Bo Yuan, Yuantao Wang, Jialin Gao, Yongrui Zhang, Yaowen Fu, Wei An

**Affiliations:** Department of Urology, The First Hospital of Jilin University, No. 71 Xinmin Street, Changchun, 130021 China

**Keywords:** Retroperitoneal laparoscopy, Radical nephrectomy, Lower pole approach, Lateroposterior space approach, Effectiveness, Safety

## Abstract

**Background:**

The objective of this study was to examine the effectiveness and safety of lower pole (LP) approach in retroperitoneal laparoscopic radical nephrectomy (LRN).

**Methods:**

One hundred thirty-two renal cancer patients were scheduled for selective retroperitoneal LRN. The surgery parameters and outcomes were compared. Out of 132 patients, 78 (59.1%) patients underwent LRN via LP approach, while 54 (40.9%) patients underwent LRN via lateroposterior space (LPS) approach.

**Results:**

Compared to LPS group, the LP group had a higher body mass index (27.0 ± 1.7 kg/m^2^ vs. 24.5 ± 1.8 kg/m^2^, *P* <  0.0001) and a larger tumor size (6.9 ± 3.5 cm vs. 4.1 ± 3.3 cm, *P* <  0.0001). The LP approach reduced the volumes of blood loss and transfusion significantly (135.3 ± 17.2 mL vs. 219.6 ± 30.9 mL, *P* <  0.0001; 55.6 ± 28.3 vs. 141.1 ± 50.4 mL, *P* <  0.0001) as compared to the LPS approach. The LP approach also decreased the risk of conversion to open procedure (1.3 vs. 7.4%, *P* <  0.05).

**Conclusions:**

The LP approach is an effective and safe alternative to the LPS approach for retroperitoneal LRN and might be more suitable for patients with obesity, large tumors, tumors located at the medial part of the kidney, or renal pedicular adhesion.

## Background

Laparoscopic radical nephrectomy (LRN) has been increasingly used as the primary surgical modality for the treatment of renal cancers [1–3] ever since Clayman et al. reported the first successful case in 1991 [4]. LRN exhibits comparable short- and long-term oncological outcomes to traditional open radical resection but is thought to be superior because of its minimal invasion [5–7]. LRN has been further modified due to the accumulation of surgical experience and improvement in laparoscopic instruments, which are used in two approaches, namely, transperitoneal access and retroperitoneal access [8]. Gaur et al. described the first case of retroperitoneal laparoscopy in radical nephrectomy using a balloon dissection technique. Both the transperitoneal approach and retroperitoneal approach result in similar surgical and oncological outcomes [9], whereas the retroperitoneal approach minimizes the technical complications associated with the peritoneal intervention and can be used in patients requiring urological surgery with a previous history of abdominal surgery [10].

The primary drawback of the retroperitoneal approach is the risk of renal vascular injury, especially in obese patients, or those with advanced tumors, renal pedicular adhesions, or refractory tumors located in proximity to the lower pole or on the dorsal side [11]. The lost control of renal vascular injury will inevitably prolong the operation and also increase the frequency of surgical morbidities and mortality. As suggested, the transperitoneal approach should be employed in those cases. Otherwise, the open procedure is required and will enhance the trauma and pain [10].

In routine retroperitoneal LRN, renal pedicular vessels are usually accessed by the lateroposterior space (LPS) approach. Using this approach, the dissection of lateroconal fascia and quadratus lumborum fascia allows the surgery entry into avascular area, or the renal LPS, margined by the quadratus lumborum, the psoas major, and Gerota’s fascia for the purpose of renal pedicular control [9]. However, the narrow space of manipulation is intrinsically subject to poor anatomical identification and a greater risk of renal pedicle injury especially in patients with obesity, larger tumors, or renal pedicle adhesions. In our practice, we attempted a new technique, the lower pole (LP) approach for the control of renal pedicular vessels in retroperitoneal LRN. In this retrospective comparative study, we examined the effectiveness and safety of the LP approach as an alternative to the LPS approach for the management of renal pedicular vessels in retroperitoneal LRN from both surgical and oncological aspects.

## Methods

### Patient enrollment

The Institutional Review Board at the First Bethune Hospital, Jilin University, approved the study protocol. One hundred forty-eight patients were consecutively enrolled into our Urology Center for radical nephrectomy (RN) from January 2008 to December 2010. Those patients were diagnosed as renal cancer based on the combination of ultrasonography, contrast-enhanced computed tomography, intravenous pyelography, and urine cytology. The patients were excluded from this study if he or she had an unresectable tumor or any distant metastases, was medically intolerable for radical resection, or was unwilling to participate in this study. The patients were well informed by an independent research nurse of the technical aspects and drawbacks of the procedures, including RN opening, transperitoneal LRN, and retroperitoneal LRN, and allowed to undergo any procedure at their own will. Informed consent was obtained from each patient before surgery.

### Preoperative workup

All renal tumors were deemed resectable. Moreover, the absence of any distant metastases was confirmed during the preoperative radiological evaluation. All patients exhibited normal renal function reserve, with a serum creatine of 44–115 μmol/L. The endogenous creatine clearance rate of the undiseased kidney ranged from 80 to 120 mL/min.

### Laparoscopic procedures

**Fig. 1:**
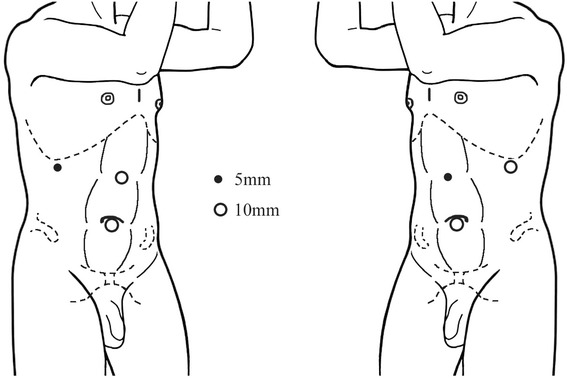
Positions of trocar ports in retroperitoneal laparoscopy (left panel, right-side laparoscopy; right panel, left-side laparoscopy)

**Fig. 2:**
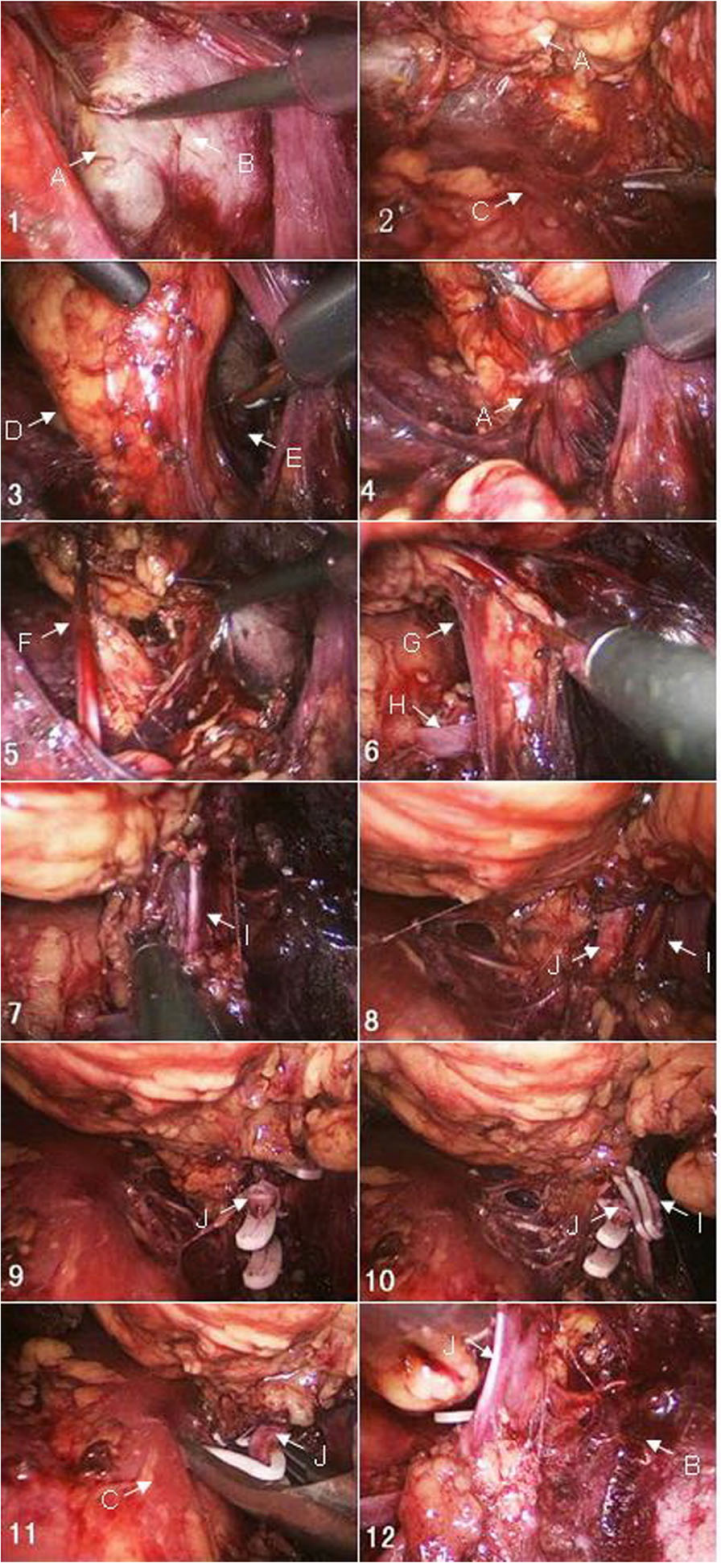
Retroperitoneal laparoscopy for radical nephrectomy using the lower pole approach. A, Gerota’s fascia; B, peritoneum; C, psoas major; D, lateroposterior space; E, lateroanterior space; F, ureter; G, connective tissue; H, lumbar vein; I, renal vein; J, renal artery

A surgical team performed all the procedures. A standard protocol of retroperitoneal (extracapsular) LRN was followed. After intubation and combined intravenous and inhalational anesthesia, the patients were placed in the standard kidney position, on the lateral side opposite to the tumor, and with the head tilted down 15° and the feet tilted down 30°. The chest and pelvis were fixed on the operative table using straps, and the wrists were cushioned with soft cloth pads. A standard three-trocar placement was used to establish access ports for either approach (Fig. 1). LPS approach was performed as previously reported [8]. LP approach was described as follows. Dissection of the renal lateroanterior space (Fig. 2 (1)) preceded that of the LPS (Fig. 2 (2)). Upon the establishment of the working space (Fig. 2 (3)), the anterior and posterior renal fasciae were transected 3.0 cm below the lower pole (Fig. 2 (4)). The ureter was mobilized (Fig. 2 (5)), and the anterior and posterior fasciae were further dissected along the inferior vena cava (IVC) for right-sided LRN or the abdominal aorta for left-sided nephrectomy. The lympho-adipose tissues between the perinephric adipose tissue and the IVC were dissected along the surface of IVC towards the renal pedicle (Fig. 2 (6)). The renal vein was dissected (Fig. 2 (7)) preliminarily to expose the renal artery located posterior (right side) or posterosuperior (left side) to the renal vein. The renal arterial sheath was further dissected to mobilize the renal artery (Fig. 2 (8)), which was interrupted using an L-size Hem-o-lok clip (Teleflex Medical, Research Triangle Park, NC) and subsequently transected (Fig. 2 (9)). The renal vein was secured using an XL-size clip and transected as well (Fig. 2 (10)). The ureter was mobilized, interrupted using an L-size clip, and transected appropriately. The upper fasciae and sub-diaphragmatic fasciae were transected to completely mobilize the diseased kidney. A concomitant ipsilateral adrenalectomy was performed in the cases of adrenal involvement, a renal tumor larger than 8 cm, a renal tumor located close to the upper pole, or a T3 tumor. The pressure of pneumoperitoneum was reduced to 4 mmHg, and the dissection surfaces were cautiously examined to exclude any active bleeding. The kidney specimen was removed using a retrieval bag (GT.K Medical, Guangzhou, China) through the laparoscope trocar incision that was extended 4–5 cm. A drain was placed onto the renal fossa through the 5-mm trocar hole. The incisions were appropriately closed using a full-thickness suture. The laparoscopy was converted to an open procedure in the case of uncontrollable bleeding or potentially massive bleeding in the dissection. A red blood cell transfusion was given to the patient with an intraoperative blood loss more than 400 mL at the discretion of the surgeon and the anesthesiologist.

### Postoperative care and follow-up

Patients were allowed to resume oral intake on the day of anal passage and start off-bed activities on the second postoperative day. The retroperitoneal drain was removed when the drainage volume fell below 10 mL daily. The patient was discharged if he or she remained uneventful following the drain removal. Patients who were pathologically diagnosed with T1–2 renal cancers received no adjuvant chemo- or radiotherapy and were regularly followed up at 3–6-month intervals for three consecutive years and at 1-year intervals afterwards. T3 patients received adjuvant medication with interleukin-2 at a quarterly or semiannual frequency for two consecutive years, at a semiannual frequency within the third year, and at an annual frequency afterwards. The follow-up tests consisted of a hepatic and renal function test, serum alkaline phosphatase, chest X-ray, and abdominal Doppler ultrasonography or computed tomography scan.

### Outcome measures

The primary outcome measures consisted of operative duration, volume of intraoperative blood loss/transfusion, rate of abdominal organ or major vessel injury, and frequency of conversion to open procedure. The secondary outcome measures included post-LRN drainage volume, time to remove the drain, and length of hospitalization.

### Statistical analysis

All the numeric data were expressed as means ± standard deviation (SD) and compared by using the Student *t* test. All the categorical data were expressed as *n* (%) and compared using Fisher’s exact probability test. A *P* value less than 0.05 was considered statistically significant.

## Results

### Baseline characteristics of patients

**Fig. 3:**
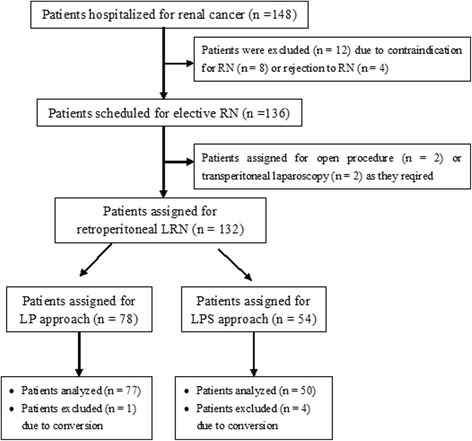
Flow chart of treatment algorithm. RN, radical nephrectomy; LRN, laparoscopic radical nephrectomy; LP, lower pole; LPS, lateroposterior space

Twelve patients were excluded from this study due to surgical contraindications (*n* = 8) or rejection for the RN procedure (*n* = 4), and four patients were assigned for open (*n* = 2) or transperitoneal (*n* = 2) LRN as the patients required. One hundred thirty-two patients were finally scheduled for elective retroperitoneal LRN. Out of 132 patients, 78 (59.1%) patients underwent LRN via LP approach and 54 (40.9%) patients underwent LRN via LPS approach (Fig. 3). Thirty-six (36/78, 46.2%) patients in the LP group underwent ipsilateral adrenalectomy compared to 19 (19/54, 35.2) patients in the LPS group (*P* > 0.05). The baseline characteristics of the two groups are shown in Table 1. The two groups were comparable in age (57.0 ± 2.1 years vs. 57.1 ± 2.1 years, *P* > 0.05) and sex (41/37 vs. 31/23, *P* > 0.05). The patients undergoing LRN via LP approach had a higher body mass index (BMI; 27.0 ± 1.7 kg/m^2^ vs. 24.5 ± 1.8 kg/m^2^, *P* <  0.0001) and a larger tumor size (6.9 ± 3.5 cm vs. 4.1 ± 3.3 cm, *P* <  0.0001) than those undergoing LRN via LPS approach. Additionally, the two groups exhibited a different location profile of the tumor (*P* <  0.05). The LP group was more frequently afflicted with a renal tumor located on the medial portion compared to the LPS group (57.7 vs. 37.0%, *P* <  0.05), whereas the LP group was less apt to have a renal tumor located at the lower pole (19.2 vs. 35.2%, *P* <  0.05). The two groups were also comparable in the patients’ previous history of abdominal surgery (3.8 vs. 1.9%, *P* > 0.05) and pre-existing medical conditions (hypertension, 25.6 vs. 22.2%, *P* > 0.05; diabetes mellitus, 25.6 vs. 18.5%, *P* > 0.05; cardiovascular diseases, 15.4 vs. 27.8%, *P* > 0.05).

**Table 1:** Baseline characteristics of patients (*n* = 132)

	LP group (*n* = 78)	LPS group (*n* = 54)	*P* value
Age (mean ± SD, year)	57.0 ± 2.1	57.1 ± 2.1	0.7884
Sex (M:F)	41/37	31/23	0.5990
BMI (mean ± SD, kg/m^2^)	27.0 ± 1.7	24.5 ± 1.5	< 0.0001
Size of tumor (mean ± SD, cm)	6.9 ± 3.5	4.1 ± 3.3	< 0.0001
Location of tumor (%)			0.0128
Upper pole	18 (23.1)	15 (27.8)	0.5471
Medial part	45 (57.7)	20 (37.0)	0.0223
Lower pole	15 (19.2)	19 (35.2)	0.0451
Previous abdominal surgery (%)	3 (3.8)	1 (1.9)	0.6443
Concomitant conditions (%)			
Hypertension	20 (25.6)	12 (22.2)	0.6851
Diabetes mellitus	20 (25.6)	10 (18.5)	0.4014
Cardiovascular diseases	12 (15.4)	15 (27.8)	0.1234

Student’s *t* test was used to analyze the data

Note: *LRN* laparoscopic radical nephrectomy, *LP* lower pole, *LPS* lateroposterior space, *BMI* body mass index

### Surgical outcomes

Surgical outcomes are shown in Table 2. LP approach appeared to shorten the operative duration significantly (125.2 ± 5.8 min vs. 135.4 ± 11.9 min, *P* <  0.0001). Moreover, the LP approach reduced the volume of blood loss and transfusion significantly compared to LPS approach (135.3 ± 17.2 mL vs. 219.6 ± 30.9 mL, *P* <  0.0001; 55.6 ± 28.3 mL vs. 141.1 ± 50.4 mL, *P* <  0.0001). Additionally, LP group had a slightly higher drainage volume compared to LPS group (225.8 ± 43.2 mL vs. 212.0 ± 22.4 mL, *P* <  0.05). Furthermore, the LP approach seemed to delay the postoperative recovery (2.1 ± 0.3 days vs. 1.8 ± 0.3 days, *P* <  0.0001), start of off-bed activities (2.6 ± 0.2 days vs. 2.3 ± 0.3 days, *P* <  0.0001), and removal of the drain (5.1 ± 0.6 days vs. 4.4 ± 0.2 days, *P* <  0.0001) compared to the LPS approach. Finally, the overall length of hospital stay was comparable between the two groups (10.7 ± 3.5 days vs. 11.4 ± 2.8 days, *P* > 0.05).

**Table 2:** Surgical outcomes of retroperitoneal LRN (*n* = 126) via LP approach compared to LPS approach (Student’s *t* test)

	LP group (*n* = 77)	LPS group (*n* = 49)	*P* value
Operative duration (mean ± SD, min)	125.2 ± 5.8	135.4 ± 11.9	< 0.0001
Volume of blood loss (mean ± SD, mL)	135.3 ± 17.2	219.6 ± 30.9	< 0.0001
Volume of transfusion (mean ± SD, mL)	55.6 ± 28.3	141.1 ± 50.4	< 0.0001
Drainage volume (mean ± SD, mL)	225.8 ± 43.2	212.0 ± 22.4	0.0331
Time to resume oral intake (mean ± SD, day)	2.1 ± 0.3	1.8 ± 0.3	< 0.0001
Time to resume off-bed activities (mean ± SD, day)	2.6 ± 0.2	2.3 ± 0.3	< 0.0001
Time of drain removal (mean ± SD, day)	5.1 ± 0.6	4.4 ± 0.2	< 0.0001
Length of hospitalization (mean ± SD, day)	10.7 ± 3.5	11.4 ± 2.8	0.2370

Note: *LRN* laparoscopic radical nephrectomy, *LP* lower pole, *LPS* lateroposterior space

### Procedural safety and complications

The two approaches had a favorable safety profile and were comparable in the frequencies of abdominal organ injury (1.3 vs. 1.9%, *P* > 0.05), retroperitoneal hematoma (1.3 vs. 3.7%, *P* > 0.05), and subcutaneous emphysema (1.3 vs. 1.9%, *P* > 0.05). However, the frequency of incidental injury of major vessels was lower in the LP group than in the LPS group (1.3 vs. 9.3%, *P* <  0.05). Of note, the LP approach also reduced the risk of conversion to the open procedure caused by the potential or uncontrollable pedicular bleeding as compared to the LPS approach (1.3 vs. 7.4%, *P* < 0.05) (Table 3). Additionally, one patient in the LPS group required conversion to transperitoneal LRN rather than open procedure due to uncontrollable bleeding from the injured renal pedicle vessels. No urinary tract infection, bleeding, renal failure, tumor dissemination, or mortality occurred following LRN via either approach.

**Table 3:** Complications and morbidities (%) of patients scheduled for retroperitoneal LRN (*n* = 132) via LP approach compared to LPS approach (Student’s *t* test)

	LP group (*n* = 78)	LPS group (*n* = 54)	*P* value
Abdominal organ injury	1/78 (1.3%)	1/54 (1.9%)	1.0000
Retroperitoneal hematoma	1/78 (1.3%)	2/54 (3.7%)	0.5672
Subcutaneous emphysema	1/78 (1.3%)	1/54 (1.9%)	1.0000
Injury of major vessels	1/78 (1.3%)	5/54 (9.3%)	0.0416
Conversion to open procedure	1/78 (1.3%)	4/54 (7.4%)	0.0416

Note: *LRN* laparoscopic radical nephrectomy, *LP* lower pole, *LPS* lateroposterior space

### Pathological outcomes and follow-up results

LRN via either approach achieved a 100% R0 resection rate, and no lymph node metastasis was detected pathologically. The pathological type and staging of renal tumors are shown in Table 4. The pathological patterns of renal tumors were comparable between the two groups (*P* > 0.05 for any specific subtype). However, the LP group exhibited a more advanced tumor profile as compared to the LPS group, less frequent pT1a tumors (6.4 vs. 31.5%, *P* < 0.0001), but more frequent pT3a tumors (38.5 vs. 11.1%, *P* < 0.0001). The two cohorts were followed up for 2–38 months, showing no local recurrence or distant metastasis. Additionally, all patients exhibited normal renal function on routine serum creatine and blood urea nitrogen tests at the follow-up visits.

**Table 4:** Pathological outcomes of patients scheduled for retroperitoneal LRN (*n* = 132) via LP approach compared to LPS approach (Student’s *t* test)

	LP group (*n* = 78)	LPS group (*n* = 54)	*P* value
Pathological type (*n*, %)			0.9910
Clear cell	56 (71.8%)	38 (70.4%)	1.0000
Granular cell	14 (17.9%)	9 (16.7%)	1.0000
Papillary	2 (2.6%)	1 (1.9%)	1.0000
Chromophobe	4 (5.1%)	3 (5.6%)	1.0000
Others	4 (5.1%)	4 (7.4%)	0.7156
Metastatic	3 (3.8%)	1 (1.9%)	0.6443
Tumor staging (*n*, %)			< 0.0001
pT1a	4 (6.4%)	17 (31.5%)	< 0.0001
pT1b	9 (11.5%)	6 (11.1%)	1.0000
pT2	31 (39.7%)	25 (46.3%)	0.4784
pT3a	30 (38.5%)	6 (11.1%)	< 0.0001
pT3b	4 (5.1%)	0 (0.0%)	0.1442

## Discussion

Vascular control of the renal pedicle is critical for LRN in either the transperitoneal or retroperitoneal approach. The rate of renal vascular morbidity is reported to be 0.4–3.5% [12, 13], which usually requires the conversion to the open procedure [11]. Transperitoneal LRN and retroperitoneal LRN are thought to be safe both surgically and oncologically, whereas the retroperitoneal approach has the advantage to reduce operative duration and minimize abdominal organ injury [11]. Therefore, this approach is more beneficial for patients with a previous history of abdominal surgery or peritoneal dialysis [10]. However, due to the restricted working space and the absence of identifiable anatomical landmarks in the retroperitoneal space [14], the retroperitoneal approach is usually restricted for tumors less than 7 cm in diameter as it is associated with a higher risk of accidental injury of the renal pedicle when larger tumors are present [10].

The LPS approach is the routine modality of renal pedicular control in retroperitoneal LRN. It allows the rapid control of renal pedicular vessels and reduces blood loss during the mobilization of the kidney. Laparoscope can be used to visualize the renal pedicle in a two- rather than three-dimensional manner in the LPS approach, which is acceptable for less obese patients or those with small tumors [15]. However, the LPS approach is less applicable in cases with excessive perinephric adipose tissue, a renal pedicle covered by the tumor located at the medial part of kidney, and peri-pedicular tissue adhesion [16]. Laparoscopic dissection is likely to cause pedicular injury and even uncontrollable hemorrhaging and thus requires the conversion to open procedure.

In the aforementioned complicated conditions, we attempt to dissect the peritoneal fascia along the peritoneum and psoas major muscle to elevate the kidney upwards from the lower pole and successfully expose the renal pedicular vessels. This approach creates a larger working space and facilitates the pedicular control. The adequate mobilization of the renal pedicle via the LP approach allows the visualization of renal pedicular vessels from both the dorsal and ventral sides by adjusting the laparoscope 30°. The three-dimensional visualization consequently maximizes the surgical field, secures the interruption and transection of the renal artery and vein, and increases the procedural safety. To the best of our knowledge, we are the first team to report this novel technique for the management of renal pedicular vessels in retroperitoneal LRN.

In our study, the LP group had a higher BMI and larger tumors as compared to the LPS group. Additionally, the LP approach reduced the volume of intraoperative blood loss, whereas the two approaches had comparable effects on post-LRN recovery. Finally, the LP approach significantly decreases the frequency of conversion to open procedure and increases the success rate of retroperitoneal LRN. In our study, the LP group had a slightly higher drainage volume compared to the LPS group; the LP approach seemed to delay the postoperative recovery, a delayed start of off-bed activities and a delayed removal of the drain compared to the LPS approach. The differences including different BMI, larger tumor size, and location might be the reasons contributing to worst surgical outcomes.

Control of renal pedicle is not only critical for reduction of renal volume and tumor size but also crucial for the prevention of hematogenous metastasis caused by stretch or compression of the tumor. A primary oncological concern is that a renal tumor located at the dorsal side may be directly pressed in the process of renal pedicular control [10]. The LP approach also offers an extra-perinephric adipose dissection in the process of mobilizing LP from the aorta or IVC, following the oncological principle of radical nephrectomy. This also reduces the direct press on the renal tumor at surgery, which therefore minimizes the likelihood of iatrogenic metastasis. No patient exhibits local recurrence or distant metastasis, and a follow-up study is ongoing to justify the long-term oncological safety of the LP approach. Nephrectomy is a destructive operation through simply cutting off the ipsilateral kidney, while not keeping the renal hilum vascular. Therefore, we did not pay attention to renal pedicle vessels. However, our experiences suggest that whether the renal pedicle vessels are easy to handle is related to the degree of obesity, the location of the tumor, and the degree of adhesion in the renal hilar tissue.

The LP technique is expected to overcome the technical drawbacks of retroperitoneal LRN and facilitates the surgical management of renal cancer patients complicated with obesity, advanced tumors, or lesions located in the medial part. Furthermore, this approach also allows the mobilization of the renal pedicle inside the lateroanterior and lateroposterior spaces simultaneously, rendering surgeons a three-dimensional visualization and manipulation of the renal tumor from both dorsal and ventral sides.

## Conclusion

The LP approach is an effective and safe alternative to the LPS approach for retroperitoneal LRN in both surgical and oncological perspectives. The LP approach results in less renal pedicular injury and has a low conversion rate. This approach is likely to be more beneficial in the case of obese patients or those with large tumors, tumors located at the medial part of kidney, or renal pedicular adhesion.

## Abbreviations

LP: Lower pole
LPS: Lateroposterior space
LRN: Laparoscopic radical nephrectomy
RN: Radical nephrectomy

## References

[1] Song S, Zhang H, Ma L (2015). The application of "renal pedicle rotation" method in retroperitoneal laparoscopic partial nephrectomy for renal ventral tumors. Journal of endourology / Endourological Society.

[2] Zhang X, Zheng T, Ma X (2005). Comparison of retroperitoneoscopic nephrectomy versus open approaches to nonfunctioning tuberculous kidneys: a report of 44 cases. J Urol.

[3] Zhu X, Yang X, Hu X, Zhang X (2016). Retroperitoneoscopic versus open surgical radical nephrectomy for 152 Chinese patients with large renal cell carcinoma in clinical stage cT2 or cT3a: a long-term retrospective comparison. J Cancer Res Ther.

[4] Clayman RV, Kavoussi LR, Soper NJ (1991). Laparoscopic nephrectomy: initial case report. J Urol.

[5] Luo JH, Zhou FJ, Xie D (2010). Analysis of long-term survival in patients with localized renal cell carcinoma: laparoscopic versus open radical nephrectomy. World J Urol.

[6] Portis AJ, Yan Y, Landman J (2002). Long-term followup after laparoscopic radical nephrectomy. J Urol.

[7] Saika T, Ono Y, Hattori R (2003). Long-term outcome of laparoscopic radical nephrectomy for pathologic T1 renal cell carcinoma. Urology.

[8] Matsumoto K, Hirayama T, Kobayashi K (2015). Laparoscopic retroperitoneal nephroureterectomy is a safe and adherent modality for obese patients with upper urinary tract urothelial carcinoma. Asian Pacific journal of cancer prevention : APJCP.

[9] Gaur DD, Agarwal DK, Purohit KC (1993). Retroperitoneal laparoscopic nephrectomy: initial case report. J Urol.

[10] Taue R, Izaki H, Koizumi T (2009). Transperitoneal versus retroperitoneal laparoscopic radical nephrectomy: a comparative study. International journal of urology : official journal of the Japanese Urological Association.

[11] Gill IS, Schweizer D, Hobart MG (2000). Retroperitoneal laparoscopic radical nephrectomy: the Cleveland clinic experience. J Urol.

[12] Barrett PH, Fentie DD, Taranger LA (1998). Laparoscopic radical nephrectomy with morcellation for renal cell carcinoma: the Saskatoon experience. Urology.

[13] Ono Y, Kinukawa T, Hattori R (1999). Laparoscopic radical nephrectomy for renal cell carcinoma: a five-year experience. Urology.

[14] Sung GT, Gill IS (2002). Anatomic landmarks and time management during retroperitoneoscopic radical nephrectomy. Journal of endourology / Endourological Society.

[15] Yang Q, Du J, Zhao ZH (2013). Fast access and early ligation of the renal pedicle significantly facilitates retroperitoneal laparoscopic radical nephrectomy procedures: modified laparoscopic radical nephrectomy. World journal of surgical oncology.

[16] Cadeddu JA, Ono Y, Clayman RV (1998). Laparoscopic nephrectomy for renal cell cancer: evaluation of efficacy and safety: a multicenter experience. Urology.

